# Life Course Trajectories of Maternal Cardiovascular Risk Factors according to Offspring Birthweight: The HUNT Study

**DOI:** 10.1038/s41598-020-66365-3

**Published:** 2020-06-26

**Authors:** Julie Horn, Eirin B. Haug, Amanda R. Markovitz, Abigail Fraser, Lars J. Vatten, Pål R. Romundstad, Janet W. Rich-Edwards, Bjørn O. Åsvold

**Affiliations:** 1 Department of Public Health and Nursing, Norwegian University of Science and Technology, Trondheim, Norway; 2 Department of Obstetrics and Gynecology, Levanger Hospital, Nord-Trøndelag Hospital Trust, Levanger, Norway; 3 K.G. Jebsen Center for Genetic Epidemiology, Department of Public Health and Nursing, Norwegian University of Science and Technology, Trondheim, Norway; 4 Department of Epidemiology, Harvard T.H. Chan School of Public Health, Boston, MA USA; 5 Division of Women’s Health, Department of Medicine, Brigham and Women’s Hospital, Boston, MA USA; 6 Mathematica Policy Research, Cambridge, MA USA; 7 MRC Integrative Epidemiology Unit and School of Social and Community Medicine, University of Bristol, Bristol, UK; 8 Department of Endocrinology, St. Olavs Hospital, Trondheim University Hospital, Trondheim, Norway

**Keywords:** Risk factors, Cardiovascular diseases, Reproductive disorders

## Abstract

Women with small or large for gestational age offspring are at increased risk of cardiovascular disease later in life. How their cardiovascular risk factors develop across the life course is incompletely known. We linked data from the population-based HUNT Study (1984–2008) and the Medical Birth Registry of Norway (1967–2012) for 22,487 women. Mixed effect models were used to compare cardiovascular risk factor trajectories for women according to first offspring birthweight for gestational age. Women with small for gestational age (SGA) offspring had 1–2 mmHg higher systolic and diastolic blood pressure across the life course, but lower measures of adiposity, compared to women with offspring who were appropriate for gestational age (AGA). In contrast, women with large for gestational age (LGA) offspring had higher measures of adiposity, ~0.1 mmol/l higher non-HDL cholesterol and triglycerides and 0.2 mmol/l higher non-fasting glucose, compared with mothers of AGA offspring. These differences were broadly stable from prior to first pregnancy until 60 years of age. Our findings point to different cardiovascular risk profiles in mothers of SGA versus LGA offspring, where giving birth to SGA offspring might primarily reflect adverse maternal vascular health whereas LGA offspring might reflect the mother’s metabolic health.

## Introduction

Pregnancy complications may inform about increased maternal risk for cardiovascular disease (CVD) later in life and be used to guide early CVD prevention efforts^1^. History of low birthweight or small for gestational age (SGA) offspring has been consistently associated with an up to two-fold increased risk of maternal CVD^2–9^. U-shaped relationships between offspring birthweight and maternal CVD mortality have also been reported, with increased CVD mortality for both mothers of SGA and large for gestational age (LGA) offspring^10,11^. To tailor preventive efforts, it is critical to understand which cardiovascular risk factors are elevated in women with SGA and LGA offspring and when in the woman’s life they develop. Several studies have examined the associations between offspring birthweight and subsequent maternal levels of CVD risk factors including body weight, blood pressure, glucose and lipids^11–15^, but no study has examined the development of these risk factors across the maternal life course.

We therefore aimed to model trajectories of commonly measured CVD risk factors by fetal growth of first offspring, using data from the Nord-Trøndelag Health Study (HUNT). We examined when in the life course differences in CVD risk factors trajectories emerge and how these differences change as women age.

## Results

Among 22,460 women, 17,995 first deliveries were AGA (80.1%), 3085 (13.7%) were SGA and 1380 were LGA (6.1%) (Table 1). Compared to women whose first offspring was AGA, women with SGA offspring were more likely to report ever daily smoking, lower educational level and use of antihypertensive medication. They were also more likely to have an earlier maternal birth year and a lower height, and a pregnancy complicated by preeclampsia, preterm birth or stillbirth. Conversely, women whose first offspring was LGA had greater height, higher educational level and a later birth year compared to women with AGA offspring, and were less likely to report ever daily smoking. LGA offspring were more often delivered preterm or postterm and LGA pregnancies were more likely to be complicated by pre-existing or gestational diabetes.

**Table 1:** Descriptive characteristics of the study population according to fetal growth in first offspring (n = 22,460).

	Fetal growth		
	SGA^a^ (n = 3,085)	AGA^b^ (n = 17,995)	LGA^c^ (n = 1,380)
**Maternal characteristics**			
Birthyear, median (IQR)	1957 (1950–1965)	1959 (1952–1968)	1961 (1953–1970)
Age at first birth, median (IQR)	23 (20–26)	23 (20–27)	24 (21–27)
Height in cm, mean (SD)	164.2 (5.8)	166.3 (5.7)	168.5 (5.8)
Ever daily smoking, n (%)			
Yes	2,096 (67.9)	10,209 (56.7)	720 (52.2)
No	989 (32.1)	7,786 (43.3)	660 (47.8)
Education, n (%)			
Lower secondary (≤9 years)	667 (21.6)	2,868 (15.9)	221 (16.0)
Upper secondary (10–12 years)	1,489 (48.3)	8,500 (47.2)	621 (45.0)
Tertiary (>12 years)	929 (30.1)	6,627 (36.8)	538 (39.0)
Ever use of antihypertensive medication, n (%)			
Yes	454 (14.7)	1,795 (10.0)	144 (10.4)
No	2,631 (85.3)	16,196 (90.0)	1,236 (89.6)
Missing	0 (0)	4 (0)	0 (0)
Age at 1st HUNT exam, median (IQR)	32 (27–38)	31 (26–37)	31 (26–36)
No. of HUNT exams, n (%)			
1	1,066 (34.6)	7,125 (39.6)	605 (43.8)
2	978 (31.7)	5,372 (29.9)	379 (27.5)
3	1,041 (33.7)	5,498 (30.6)	396 (28.7)
HUNT exams relative to first pregnancy, n (%)			
Before first pregnancy only	235 (7.6)	1,660 (9.2)	158 (11.5)
After first pregnancy only	2,588 (83.9)	14,526 (80.7)	1,078 (78.1)
Before and after first pregnancy	262 (8.5)	1,809 (10.1)	144 (10.4)
Achieved parity			
1	455 (14.8)	2,231 (12.4)	195 (14.1)
2	1,401 (45.4)	8,147 (45.3)	610 (44.2)
3	920 (29.8)	5,762 (32.0)	436 (31.6)
≥4	309 (10.0)	1,855 (10.3)	139 (10.1)
**First pregnancy characteristics**			
Pre-pregnancy diabetes, n (%)	4 (0.1)	38 (0.2)	9 (0.6)
Gestational diabetes, n (%)	1 (0.03)	36 (0.2)	9 (0.7)
Pre-pregnancy hypertension, n (%)	13 (0.4)	58 (0.3)	5 (0.4)
Preeclampsia or gestational hypertension, n (%)	345 (11.2)	1,027 (5.7)	96 (7.0)
Gestational length, n (%)			
Preterm (<37 weeks)	238 (7.7)	868 (4.8)	162 (11.7)
Term (37–41 weeks)	2,374 (77.0)	14,270 (79.3)	963 (69.8)
Postterm (≥42 weeks)	473 (15.3)	2,857 (15.9)	255 (18.5)
Birthweight in grams, mean (SD)	2,715 (429)	3,510 (439)	4,306 (540)
Stillbirths, n (%)	76 (2.5)	86 (0.5)	8 (0.6)

SGA = small for gestational age; AGA = appropriate for gestational age; LGA = large for gestational age; IQR = interquartile range, SD = standard deviation. Percentages may not sum to 100% due to rounding.

^a^SGA was defined as birthweight below the 10^th^ centile for the Norwegian population.

^b^AGA was defined as birthweight ≥10^th^ and ≤90^th^ centile for the Norwegian population.

^c^LGA was defined as birthweight above the 90^th^ centile for the Norwegian population.

### SGA

**Figure 1:**
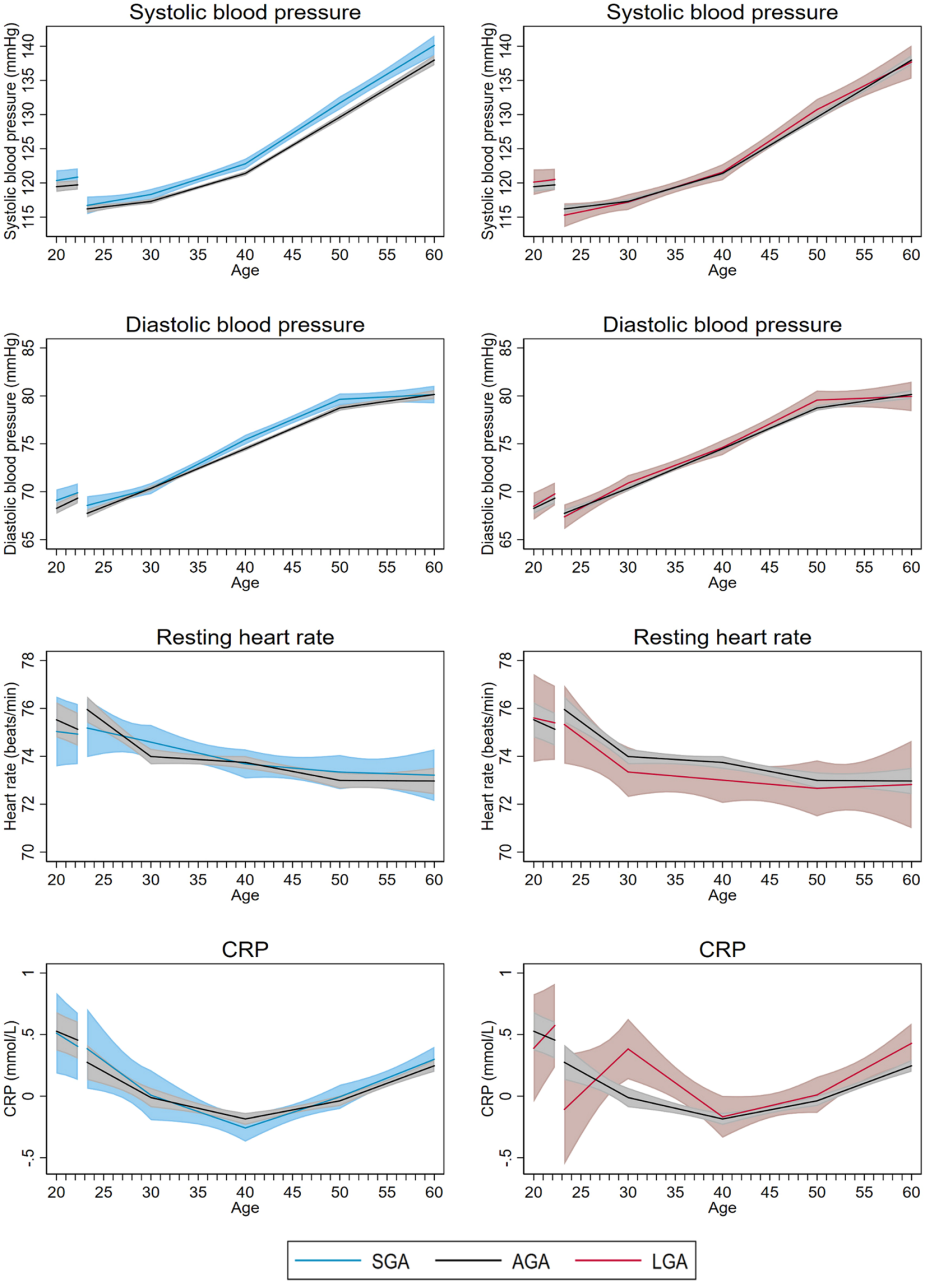
Life course trajectories of mean systolic blood pressure, diastolic blood pressure, resting heart rate, and CRP for women according to weight for gestational age in first pregnancy.

Women with SGA offspring had different blood pressure trajectories than women with AGA offspring (LRT: p < 0.0001 for systolic and p < 0.01 for diastolic blood pressure). Already at age 20, before first pregnancy, though not yet statistically significant, systolic and diastolic blood pressure appeared to be slightly higher among women who later gave birth to SGA offspring (Fig. 1, Table 2). Throughout middle age, systolic and diastolic blood pressure was consistently 1–2 mmHg higher in women with SGA compared with AGA offspring. The difference in systolic blood pressure was still present at age 60 (2.1 mmHg, 95%CI: 0.6, 3.7), whereas the difference in diastolic blood pressure attenuated from 50 to 60 years of age (Fig. 1, Table 2). These differences in blood pressure, however, did not translate into statistically significantly higher risk of hypertension, except at ages 40 and 50 (Fig. S2, Table 3).

**Table 2:** Cardiovascular risk factors by age at follow-up in women according to weight for gestational age in first offspringa.

Cardiovascular risk factors	SGA^b^ (n = 3,085)		AGA^c^ (n = 17,995)	LGA^d^ (n = 1,380)	
	Difference between SGA and AGA estimates (95% CI)	p-value	Predicted mean (95% CI)	Difference between LGA and AGA estimates (95% CI)	p-value
**Systolic blood pressure (mmHg)**					
20 years	0.9 (−0.6, 2.4)	0.24	119.5 (118.7, 120.2)	0.7 (−1.2, 2.5)	0.49
30 years	1.0 (0.2, 1.9)	0.02	117.3 (116.9, 117.7)	−0.1 (−1.3, 1.1)	0.87
40 years	1.4 (0.6, 2.1)	0.001	121.4 (121.1, 121.7)	0.2 (−1.0, 1.4)	0.78
50 years	2.1 (1.1, 3.1)	<0.001	129.6 (129.2, 130.1)	1.1 (−0.5, 2.7)	0.18
60 years	2.1 (0.6, 3.7)	0.006	138.0 (137.2, 138.7)	−0.3 (−2.8, 2.2)	0.82
**Diastolic blood pressure (mmHg)**					
20 years	0.8 (−0.3, 2.0)	0.15	68.3 (67.7, 68.8)	0.2 (−1.2, 1.6)	0.76
30 years	−0.03 (−0.6, 0.6)	0.93	70.4 (70.1, 70.6)	0.6 (−0.3, 1.4)	0.21
40 years	1.0 (0.4, 1.5)	0.001	74.5 (74.3, 74.7)	0.1 (−0.7, 0.9)	0.76
50 years	0.9 (0.3, 1.6)	<0.01	78.7 (78.5, 79.0)	0.8 (−0.2, 1.9)	0.12
60 years	−0.01 (−1.0, 1.0)	0.98	80.2 (79.7, 80.6)	−0.2 (−1.8, 1.4)	0.80
**BMI (kg/m**^**2**^**)**					
20 years	−0.3 (−0.5, −0.2)	<0.001	22.8 (22.7, 22.9)	1.1 (0.9, 1.3)	<0.001
30 years	−0.4 (−0.6, −0.2)	<0.001	24.0 (23.9, 24.1)	1.3 (1.0, 1.6)	<0.001
40 years	−0.6 (−0.8, −0.4)	<0.001	25.0 (24.9, 25.1)	1.3 (1.0, 1.6)	<0.001
50 years	−0.6 (−0.8, −0.4)	<0.001	26.1 (26.0, 26.2)	1.4 (1.1, 1.8)	<0.001
60 years	−0.7 (−1.1, −0.4)	<0.001	26.8 (26.6, 27.0)	1.4 (1.0, 1.9)	<0.001
**Waist circumference (cm)**					
20 years	−1.0 (−2.6, 0.5)	0.18	77.0 (76.3, 77.7)	3.4 (1.7, 5.1)	<0.001
30 years	−0.5 (−1.4, 0.3)	0.21	81.8 (81.4, 82.1)	3.5 (2.3, 4.7)	<0.001
40 years	−1.5 (−2.1, −0.8)	<0.001	83.6 (83.4, 83.9)	3.0 (2.1, 3.9)	<0.001
50 years	−1.4 (−2.1, −0.7)	<0.001	85.8 (85.5, 86.1)	2.3 (1.3, 3.4)	<0.001
60 years	−1.3 (−2.2, −0.4)	<0.001	87.1 (86.7, 87.6)	3.3 (1.9, 4.8)	<0.001
**Hip circumference (cm)**					
20 years	−1.5 (−2.8, −0.1)	0.04	97.9 (97.3, 98.6)	2.7 (1.2, 4.3)	0.001
30 years	−0.8 (−1.6, −0.1)	0.03	100.9 (100.6, 101.2)	2.9 (1.9, 4.0)	<0.001
40 years	−1.5 (−2.0, −1.0)	<0.001	102.1 (101.9, 102.3)	2.3 (1.5, 3.0)	<0.001
50 years	−1.1 (−1.6, −0.5)	<0.001	103.2 (103.0, 103.4)	1.8 (1.0, 2.6)	<0.001
60 years	−1.2 (−1.9, −0.5)	0.001	103.2 (102.9, 103.6)	1.3 (0.2, 2.4)	0.03
**Waist to hip ratio**					
20 years	0.0 (−0.01, 0.01)	0.54	0.8 (0.8, 0.8)	0.01 (0.00–0.03)	0.01
30 years	0.0 (−0.01, 0.01)	0.98	0.8 (0.8, 0.8)	0.01 (0.00–0.02)	0.003
40 years	0.0 (−0.01, 0.00)	0.06	0.8 (0.8, 0.8)	0.01 (0.00–0.02)	<0.001
50 years	−0.01 (−0.01, 0.00)	0.02	0.8 (0.8, 0.8)	0.01 (0.00–0.01)	0.03
60 years	0.0 (−0.01, 0.01)	0.87	0.8 (0.8, 0.8)	0.02 (0.01–0.03)	<0.001
**Non-HDL cholesterol (mmol/l)**					
20 years	0.1 (−0.1, 0.2)	0.51	3.2 (3.1, 3.3)	0.1 (−0.1, 0.2)	0.41
30 years	0.1 (−0.04, 0.1)	0.32	3.4 (3.4, 3.4)	0.1 (0.01, 0.3)	0.03
40 years	−0.01 (−0.1, 0.1)	0.78	3.7 (3.7, 3.8)	0.1 (0.01, 0.2)	0.03
50 years	−0.1 (−0.1, 0.02)	0.18	4.3 (4.3, 4.3)	0.1 (0.04, 0.2)	0.01
60 years	−0.04 (−0.1, 0.1)	0.34	4.8 (4.7, 4.8)	0.1 (−0.1, 0.3)	0.18
**HDL cholesterol (mmol/l)**					
20 years	0.01 (−0.1, 0.1)	0.85	1.4 (1.4, 1.5)	−0.1 (−0.1, 0.0)	0.04
30 years	0.00 (−0.03, 0.03)	0.91	1.4 (1.4, 1.4)	−0.03 (−0.1, 0.01)	0.17
40 years	0.02 (−0.01, 0.04)	0.15	1.4 (1.4, 1.4)	−0.02 (−0.1, 0.01)	0.14
50 years	0.01 (−0.02, 0.03)	0.65	1.5 (1.5, 1.5)	−0.01 (−0.04, 0.02)	0.55
60 years	0.02 (−0.01, 0.04)	0.28	1.5 (1.5, 1.6)	−0.04 (−0.1, 0.00)	0.06
**Triglycerides (mmol/l)**					
20 years	0.02 (−0.1, 0.1)	0.69	1.2 (1.2, 1.3)	0.02 (−0.1, 0.1)	0.74
30 years	0.01 (−0.1, 0.1)	0.82	1.2 (1.1, 1.2)	0.1 (−0.01, 0.2)	0.09
40 years	0.01 (−0.04, 0.1)	0.62	1.2 (1.2, 1.3)	0.1 (0.01, 0.2)	0.02
50 years	0.02 (−0.04, 0.1)	0.49	1.5 (1.4, 1.5)	0.1 (−0.03, 0.2)	0.21
60 years	−0.03 (−0.1, 0.1)	0.56	1.7 (1.6, 1.7)	0.1 (−0.1, 0.2)	0.21
**Non-fasting glucose (mmol/l)**					
20 years	−0.03 (−0.2, 0.1)	0.69	4.9 (4.7, 5.0)	0.03 (−0.2, 0.2)	0.75
30 years	−0.05 (−0.2, 0.04)	0.28	4.9 (4.8, 4.9)	0.2 (0.1, 0.3)	0.01
40 years	0.00 (−0.1, 0.1)	0.99	5.1 (5.1, 5.2)	0.1 (−0.1, 0.2)	0.33
50 years	−0.04 (−0.1, 0.04)	0.30	5.3 (5.2, 5.4)	0.2 (0.1, 0.4)	0.001
60 years	−0.1 (−0.2, 0.04)	0.18	5.5 (5.4, 5.6)	0.2 (−0.03, 0.4)	0.10
**Resting heart rate (beats/min)**					
20 years	−0.5 (−1.9, 1.0)	0.51	75.5 (74.8, 76.3)	0.1 (−1.8, 1.9)	0.93
30 years	0.6 (0.2, 1.4)	0.12	74.0 (73.7, 74.3)	−0.7 (−1.7, 0.4)	0.24
40 years	−0.1 (−0.7, 0.6)	0.85	73.7 (73.5, 74.0)	−0.7 (−1.7, 0.2)	0.14
50 years	0.4 (−0.4, 1.1)	0.37	73.0 (72.7, 73.3)	−0.3 (−1.5, 0.9)	0.59
60 years	0.2 (−0.9, 1.4)	0.67	73.0 (72.4, 73.5)	−0.2 (−2.0, 1.1)	0.88
**CRP**^**e**^ **(mg/l)**					
20 years	1.0 (0.7, 1.4)	0.93	1.7 (1.5, 2.0)	0.8 (0.6, 1.3)	0.53
30 years	1.0 (0.8, 1.3)	0.86	1.0 (0.9, 1.1)	1.5 (1.2, 1.9)	0.002
40 years	0.9 (0.8, 1.1)	0.23	0.8 (0.8, 0.9)	1.0 (0.9, 1.2)	0.85
50 years	1.0 (0.9. 1.2)	0.55	1.0 (0.9, 1.0)	1.1 (0.9, 1.2)	0.53
60 years	1.1 (1.0, 1.2)	0.35	1.3 (1.2, 1.3)	1.2 (1.0, 1.4)	0.03

SGA = small for gestational age; AGA = appropriate for gestational age; LGA = large for gestational age; CI = confidence interval; BMI = body mass index; HDL = high density lipoprotein; CPR = C-reactive protein.

^a^Given as predicted mean levels for women with AGA offspring and mean differences (SGA-AGA or LGA-AGA) for women with SGA and LAG offspring. All estimates are adjusted for age at measurement, maternal height, smoking status, educational status, HUNT survey and age at first birth.

^b^SGA was defined as birthweight below the 10^th^ centile for the Norwegian population.

^c^AGA was defined as birthweight ≥10^th^ and ≤90^th^ centile for the Norwegian population.

^d^LGA was defined as birthweight above the 90^th^ centile for the Norwegian population.

^e^CRP is given as geometric mean values where the difference equates to the ratio of geometric mean CRP between women with SGA or LGA offspring and women with AGA offspring.

**Table 3:** Predicted proportions of hypertension, obesity and diabetes by age at follow-up in women according to weight for gestational age in first offspring.

	Predicted proportion among women with AGA^b^ offspring		Difference between SGA^c^ and AGA proportions		Difference between LGA^d^ and AGA proportions	
	Percent	(95% CI)	Percentage points	(95% CI)	Percentage points	(95% CI)
**Hypertension**						
20 years	5.8	(4.6, 7.4)	2.4	(−1.1, 6.0)	0.4	(−3.6, 4.4)
30 years	5.8	(5.2, 6.4)	0.7	(−0.8, 2.1)	−0.4	(−2.3, 1.6)
40 years	13.5	(12.6 ‒ 14.4)	2.3	(0.2, 4.4)	1.0	(−2.1, 4.2)
50 years	33.6	(32.0 ‒ 35.2)	4.9	(1.4, 8.5)	3.1	(−2.5, 8.7)
60 years	57.6	(54.9 ‒ 60.2)	3.6	(−1.2, 8.4)	5.6	(−2.2, 13.3)
**Obesity**						
20 years	2.9	(2.3, 3.5)	−0.6	(−1.6, 0.4)	5.0	(1.9, 7.9)
30 years	6.8	(6.0, 7.7)	−0.04	(−1.6, 1.5)	4.7	(1.9, 7.4)
40 years	8.5	(7.7, 9.3)	−1.8	(−3.1, −0.6)	5.2	(2.5, 7.9)
50 years	9.7	(8.8, 10.7)	−2.1	(−3.5, −0.7)	3.3	(0.4, 6.2)
60 years	11.6	(10.3, 13.2)	−1.6	(−3.8, 1.0)	2.6	(−1.9, 7.0)
**Diabetes**						
20 years	0.4	(0.1, 1.0)	−0.2	(−0.8, 0.5)	0.1	(−0.8, 1.0)
30 years	0.4	(0.2, 0.6)	−0.2	(−0.5, 0.03)	1.6	(0.3, 2.9)
40 years	0.9	(0.7, 1.1)	−0.4	(−0.9, 0.04)	1.8	(0.4, 3.3)
50 years	1.5	(1.1, 1.9)	−0.3	(−1.0, 0.3)	1.3	(−0.3, 2.9)
60 years	3.4	(2.5, 4.4)	−0.9	(−2.3, 0.6)	1.9	(−1.5, 5.4)

^a^Population average proportions are estimated with all covariates set at their means and as if the woman has her first birth at age 23. All estimates are adjusted for age at measurement, maternal height, smoking status, educational status, HUNT survey and age at first birth.

^b^AGA was defined as birthweight ≥10^th^ and ≤90^th^ centile for the Norwegian population.

^c^SGA was defined as birthweight below the 10^th^ centile and for the Norwegian population.

^d^LGA was defined as birthweight above the 90^th^ centile for the Norwegian population.

**Figure 2:**
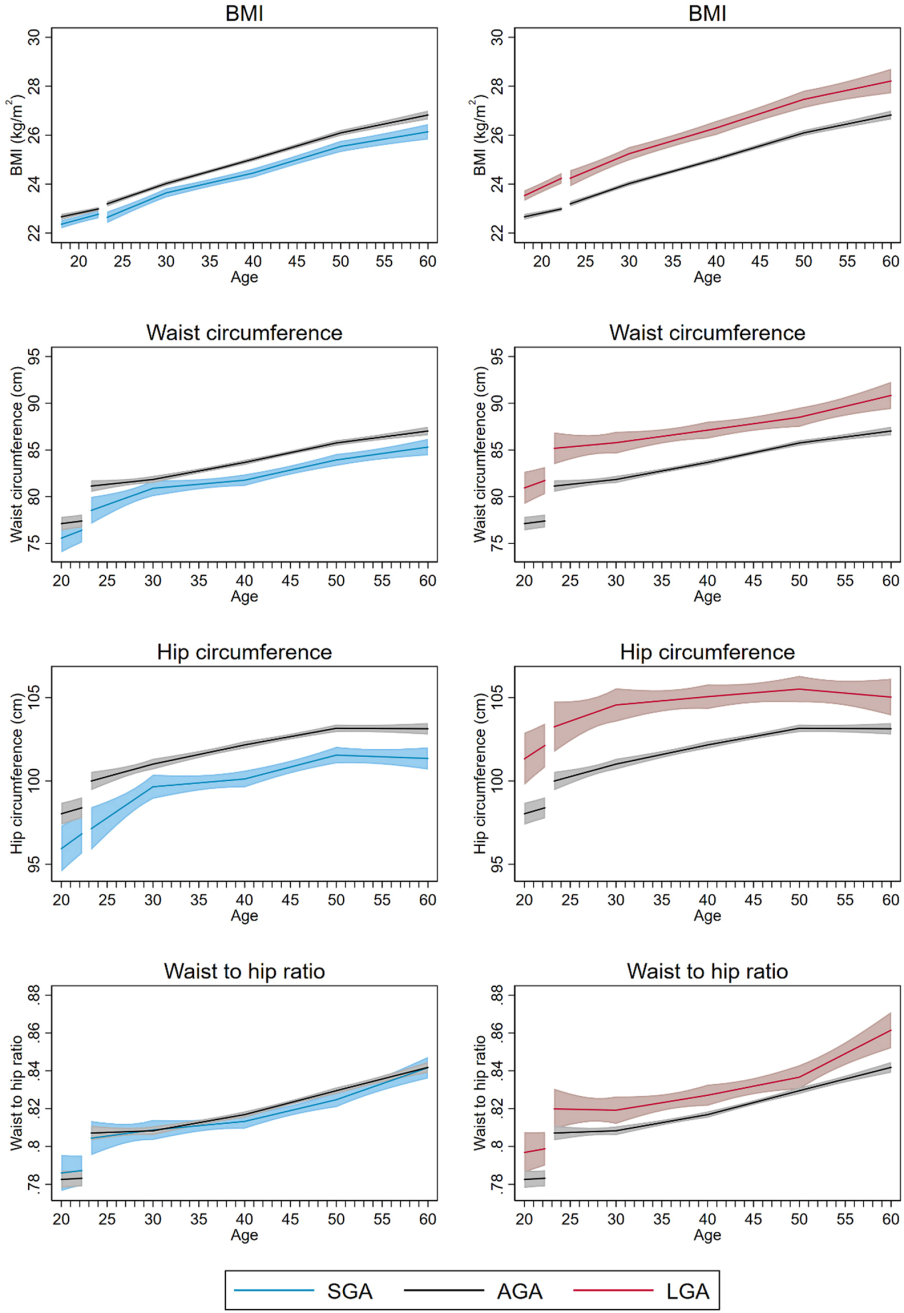
Life course trajectories of mean BMI, waist circumference, hip circumference, and waist to hip ratio for women according to weight for gestational age in first pregnancy.

**Figure 3:**
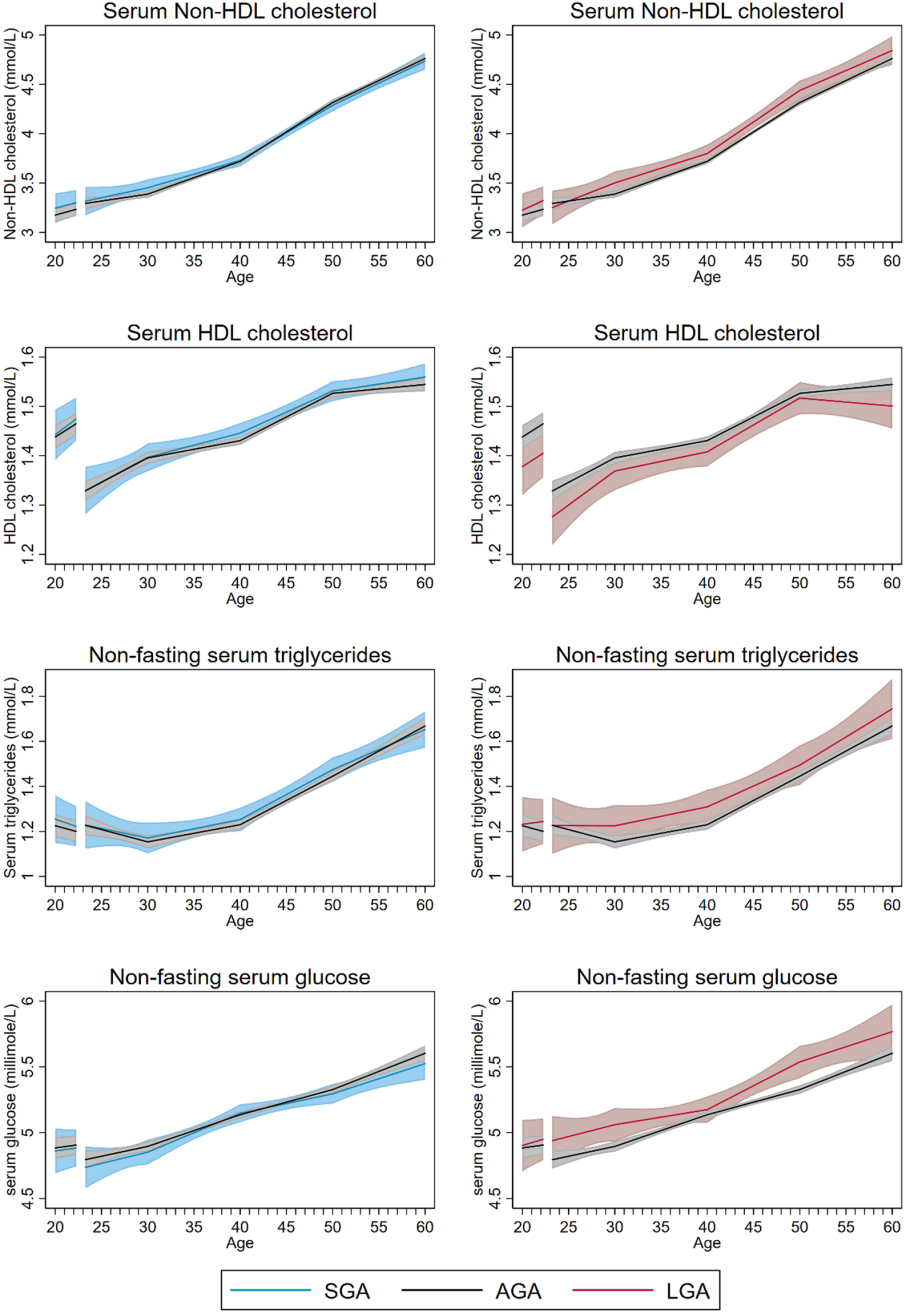
Life course trajectories of mean nonfasting serum Non-HDL and HDL cholesterol, triglycerides, and glucose for women according to weight for gestational age in first pregnancy.

Measures of adiposity, including BMI, waist and hip circumference, were lower in women with SGA compared with AGA offspring throughout the entire age span (Fig. 2, Table 2) (LRT: p < 0.0001 for all measures of adiposity). At age 60, the mean difference was −0.7 kg/m^2^ (95% CI: −1.1, −0.4) for BMI, −1.3 cm (95% CI: −2.2, −0.4) for waist circumference and −1.2 cm (95% CI: −1.9, −0.5) for hip circumference (Table 2). By age 60, the estimated prevalence of obesity was 1.6 percentage points (95% CI: −3.8, 1.0) lower in women with SGA than in women with AGA infants (Fig. S2, Table 3). Trajectories of serum lipids, glucose, CRP and resting heart rate were largely similar for women with SGA and AGA infants (Table 2, Figs. 1 and 3).

### LGA

Blood pressure trajectories for women with LGA and AGA offspring were largely similar (Fig. 1) (LRT: p = 0.12 for systolic and p = 0.49 for diastolic blood pressure). Women with LGA offspring had higher measures of adiposity throughout the entire age span compared to women with AGA (LRT: p < 0.0001 for all measures of adiposity) (Fig. 2, Table 2). At age 60, the mean difference was 1.4 kg/m^2^ (95% CI: 1.0, 1.9) for BMI, 3.3 cm (95% CI: 1.9, 4.8) for waist circumference and 1.3 cm (95% CI: 0.2, 2.4) for hip circumference (Table 2). At age 60, the estimated prevalence of obesity was 2.6 percentage points (95% CI: −1.9, 7.0) higher than in women with AGA (Table 3). Women with LGA offspring also had less favorable lipid and glucose trajectories from 30 years of age and onwards (LRT: p = 0.01 for non-HDL cholesterol; p = 0.02 for triglycerides and p < 0.0001 for non-fasting glucose), but the differences between women with LGA and AGA offspring were minor, ~0.1 mmol/l for non-HDL cholesterol and triglycerides and 0.2 mmol/l for non-fasting glucose (Fig. 3, Table 2). The prevalence of diabetes was higher in women with LGA offspring throughout the entire age span, with 1.9 percentage points (95% CI: −1.5, 5.4) higher prevalence at age 60 (Fig. S2, Table 3).

In sensitivity analyses, women whose first offspring was extremely SGA, defined as birthweight <3^rd^ percentile, had higher systolic and diastolic blood pressure compared to women with offspring birthweight between the 3^rd^ and the 97^th^ percentile (Table S2). These differences in blood pressure where higher than those observed in our main analyses where we compared women with SGA offspring <10^th^ percentile to women with AGA offspring. Likewise, women whose first offspring was extremely LGA, defined as birthweight >97^th^ percentile, had higher anthropometric measures, higher non-HDL cholesterol and lower HDL cholesterol than women with offspring birthweight between 3^rd^ and 97^th^ percentile and these differences were higher than those observed in our main analysis between women with LGA offspring >90^th^ percentile and AGA offspring (Table 2, Table S2).

After restricting our analyses to live births not complicated by HDP or diabetes, the differences in blood pressure trajectories between women with SGA and AGA offspring were smaller than in the main analysis (Table S3). However, differences in trajectories of CVD risk factors between women with LGA and AGA offspring were similar to those observed in the main analysis (Table S3).

Additional analyses among the subset of women with available information on offspring birthweight in first and second pregnancy indicated that women with SGA or LGA offspring in both first and second pregnancy generally had a more adverse cardiovascular risk profile than women with one SGA or LGA offspring (Figs. S3–S5).

## Discussion

In this first study of maternal life course trajectories of cardiovascular risk factors according to offspring birthweight, blood pressure was higher throughout adulthood in women with SGA offspring. In contrast, women with LGA offspring had more adiposity and less favorable lipid and glucose measures from prior to first birth until beyond menopause.

The large study size and the long follow-up period in a stable and homogenous population of women enabled us to examine CVD risk factors by offspring growth with high precision throughout a woman’s life course. We examined a broad range of clinically measured CVD risk factors and included repeated measurements of risk factors as well as measurements both before and after first pregnancy. We were able to adjust for maternal height, smoking and educational status in order to reduce the influence of these factors on the risk factor trajectories. We also adjusted for time since last meal since glucose and lipids were measured in non-fasting blood samples. Another strength is the high validity of registry-based information on birthweight for gestational age, HDP^16,17^ and pre-pregnancy diabetes, whereas GDM is likely underreported in the MBRN for the time period covered in our study^18^.

During the past decades, birthweight and BMI increased in Norway while blood pressure, heart rate and total cholesterol levels decreased^19,20^. We therefore adjusted for age and HUNT survey to reduce the influence of these secular trends on the risk factor trajectories. The use of antihypertensive medication may have affected the shape of our blood pressure trajectories. However, we added recommended constants to observed blood pressure measurements for women using antihypertensive medication in order to reduce misclassification errors^21,22^. Data on use of lipid lowering medication were not available and we therefore could not assess the possible influence of lipid lowering medication on the shape of the lipid trajectories. More than the expected 10 percent of pregnancies in our study population delivered from 1967–2012 were defined SGA and less than the expected 10 percent of pregnancies were defined LGA using a Norwegian sex- and gestational age-specific population reference based on the period 1987–98^20^. Explanations for the higher proportion of SGA and lower proportion of LGA offspring compared to the reference population, include the increase in birthweight during the time period of our study as well as lower birthweight among first born offspring compared to offspring born to parous women^20,23^.

The population of Nord-Trøndelag is fairly representative of Norway as a whole^24^, and our results are likely generalizable to other European populations. However, the association between offspring weight and maternal risk factors for CVD might vary according to race^14,15^ and our findings may therefore not be generalizable to more ethnically diverse populations.

Our work builds on earlier studies that examined the association between offspring birthweight and CVD risk factors using data from HUNT2^12,13^. In contrast to this previous work, we were able to assemble maternal CVD risk factor trajectories throughout a woman’s lifespan and examine when in life differences in CVD risk factors were established and how these differences developed with age.

Although no previous study has estimated CVD risk factors trajectories after delivery of SGA or LGA offspring, several studies included single observations of CVD risk factors and found less adiposity and higher blood pressure^9,15^ and prevalence of hypertension^11,14,25^ among mothers of SGA or low birthweight offspring. Our finding of systolic blood pressure differences persisting beyond menopausal age is consistent with a study that examined cardiovascular risk factors according to first offspring birthweight in women older than 70 years^15^. That study did not include pregnancies complicated by HDP and thus supports our observation that the association between SGA offspring and higher maternal blood pressure is not solely driven by HDP. The differences in blood pressure in middle age between women with and without SGA offspring were comparable to those reported in a Norwegian cohort study^9^, whereas blood pressure differences among older women in the present study were lower compared to those reported in a US study population^15^. We did not confirm previous reports of increased diabetes risk among women with low birthweight offspring^11,26–28^. However, most of those studies examined birthweight unadjusted for gestational age. We used birthweight standardized for gestational age and sex that enabled us to narrow our investigation to fetal growth instead of gestation length^10^.

Higher offspring birthweight has consistently been associated with adverse maternal metabolic factors in pregnancy including higher BMI^29,30^ and glucose^31^ and less favorable lipid levels^32,33^. Our results suggest that these differences in CVD risk factors persist into older age and are supported by a recent Norwegian cohort study, also including a small proportion of our study participants, that reported higher BMI and triglyceride levels among middle aged women with higher offspring birthweight^9^. In accordance with our results, previous studies consistently found higher offspring birthweight to be associated with increased risk of maternal diabetes^11,26,27,34,35^ or diabetes mortality^28^. Cohort studies of the association between high offspring birthweight and hypertension have found either no association^9^ or an U-shaped risk profile of hypertension with respect to offspring birthweight^11^.

While women regardless of offspring birthweight may lower their long-term risk of CVD by lifestyle modifications, the results of our study suggest that women with SGA offspring may especially benefit from postpartum advice regarding prevention of hypertension. On the other hand, diet modification and exercise aimed at weight loss might be more relevant for women with LGA offspring. The postpartum period has been described as a “window of opportunity” for lifestyle modifications and given that the differences in cardiovascular risk factors already emerged prior to pregnancy, early preventive efforts may help to reduce the excess risk of CVD in women with SGA or LGA offspring.

In conclusion, our findings provide a comprehensive picture of the long-term association between offspring birthweight and maternal CVD risk factors. Compared to women with AGA offspring, mothers of SGA offspring had higher blood pressure whereas mothers of LGA offspring had less favorable anthropometric, lipid and glucose levels throughout their life course. Our findings point to different cardiovascular risk profiles in mothers of SGA versus LGA offspring, where giving birth to SGA offspring may primarily reflect adverse maternal vascular health whereas LGA offspring may reflect the mother’s metabolic health.

## Methods

### Study population

We linked information from the Medical Birth Registry of Norway (MBRN) and the HUNT Study using the unique identification number of Norwegian residents. The MBRN has collected data on all deliveries in Norway through standardized forms since 1967, including information on the mother’s and child’s health and complications during pregnancy and childbirth^36^.

The HUNT Study is a population-based open cohort study to which all residents of Nord-Trøndelag county, Norway, aged ≥20 years, are invited. The HUNT Study includes information on participants’ general health and life style factors collected by questionnaires, interviews, clinical measurements and non-fasting blood samples.

Our study population includes 25,932 women who had their first birth recorded in the MBRN between 1967 and 2012 and who participated in one or more of the first three surveys of the HUNT Study: HUNT1 (1984–86)^37^, HUNT2 (1995–97)^38^ and HUNT3 (2006–08)^24^. We excluded 1,972 women because of the following characteristics of their first birth: multiple birth (n = 314), gestational age below 22 or above 44 weeks (n = 240), birthweight below 500 g (n = 31), missing data on gestational age, offspring sex or birthweight (n = 1,323) or unlikely combination of birthweight and gestational age, defined as birthweight for gestational age z-score of < −4.0 or >4.0 (n = 64). We excluded from our analyses measurements of cardiovascular risk factors that were performed during pregnancy or up to 3 months postpartum, resulting in the additional exclusion of 328 women. Finally, we excluded 1,172 women with missing information on smoking status or educational level, leaving 22,460 women in the final study population (Fig. S1).

### Exposure assessment

We calculated z-scores of birthweight for sex and gestational age using standards estimated from the MBRN for the period 1987–1998^20^. Until 1998, gestational age was determined by first day of last menstrual period. Afterwards, if available, gestational age was estimated by ultrasound examination in the second trimester that was performed in approximately 98% of women^39^.

SGA was defined as birthweight z-score below the 10^th^ percentile and LGA was defined as birthweight z-score above the 90^th^ percentile for the Norwegian population^20^. All other births were defined as appropriate for gestational age (AGA). As a sensitivity analysis, we studied severe SGA, defined as birthweight z-score below the 3^rd^ percentile, to better differentiate between growth restricted and physiologically small newborns^40^. Likewise, we defined severe LGA as birthweight z-score above the 97^th^ centile. In this sensitivity analysis, AGA was defined as birthweight between the 3^rd^ and the 97^th^ percentile.

### Outcome assessment

A detailed description of cardiovascular risk factor measurement methods in HUNT has been given previously^41^ and is also summarized in supplemental table S1. Briefly, the clinical examinations were performed by trained staff and included measurement of blood pressure, heart rate, height, weight, waist and hip circumference. Blood samples were analyzed for non-fasting serum lipids, glucose and C-reactive protein (CRP). Questionnaires assessed information on diagnosis of diabetes, use of antihypertensive medication, and, in HUNT3 only, self-reported weight and height at age 18. Obesity was defined as body mass index (BMI) ≥ 30 kg/m^2^. In 1,978 participants taking antihypertensive medication, we added 10 mmHg to the systolic and 5 mmHg to the diastolic blood pressure measurements to approximate the values they would have had without treatment^21,22^. Hypertension was defined as systolic blood pressure ≥140 mmHg, diastolic blood pressure ≥90 mmHg or self-report of antihypertensive medication. Diabetes mellitus was either self-reported or defined by elevated glucose measures (Table S1). The distribution of observations by time since first birth and HUNT survey is given in Fig. S6.

### Covariates

The MBRN provided information on maternal age at first birth (AFB). HUNT questionnaires assessed information on smoking status (ever or never daily smoking), educational level (lower secondary education (<10 years), upper secondary education (10–12 years) or tertiary education (>12 years)) and time since last meal (<1, 1, 2, 3, 4, 5 or ≥6 hours). Information on educational level was collected in HUNT1 and HUNT2, but not in HUNT3 where we derived educational level from work titles collected by interview based on recommendations from Statistics Norway^42^.

### Statistical analysis

We used linear spline mixed-effects models to model life course trajectories of cardiovascular risk factors for women with SGA, AGA and LGA first offspring^43^. A series of linear splines with knot points at 10-year intervals allowed us to model a non-linear relationship between age and CVD risk factors. Random intercepts and slopes accounted for up to 3 repeated measurements per woman for most risk factors and up to 4 repeated measurements for BMI^44^. Furthermore, we incorporated two variables to account for the changes in risk factor trajectories associated with first birth: one for the immediate change in CVD risk factor level from pre-pregnancy until 3 months after first birth and another one for the change in CVD risk factor progression after 3 months postpartum (slope). To further increase flexibility of the trajectories we included interaction terms between these two variables and fetal growth (SGA, LGA), maternal AFB, educational level and smoking status. All models were adjusted for maternal height, smoking status, highest obtained educational status, HUNT survey and AFB. Analyses of glucose and triglycerides were additionally adjusted for time since last meal. For CRP, we log-transformed the values due to a skewed distribution and used a linear spline regression model with a cluster-robust estimate of variance (Huber/White sandwich estimate) to model the trajectories due to a limited number of repeated measurements. All analyses were conducted with Stata IC 14 (StataCorp, College Station, Texas, USA) and MLwiN version 2.34^45^ via the runmlwin^46^ command in Stata.

We predicted cardiovascular risk factors using the above described models and contrasted risk factor trajectories for women whose first newborn was SGA, AGA or LGA. To graph the trajectories, AFB was set to age 23 (median AFB in our study population) and all other covariates were set to their mean. Cardiovascular risk factor trajectories are shown up to age 60 due to sparse data for older women. We were not able to model risk factor trajectories during pregnancy and shortly after birth due to limited data and thus added a gap in the risk factor trajectories. We performed likelihood ratio tests (LRT) to examine whether risk factor trajectories differed by fetal growth by comparing models with and without parameters related to SGA or LGA, respectively. To estimate the probabilities of obesity, hypertension and diabetes mellitus, we used logistic regression with cluster-robust variance.

As a sensitivity analysis, we examined if severe SGA or LGA was associated with a more adverse cardiovascular risk profile. In addition, to explore whether differences in trajectories were largely driven by pregnancy complications that may co-occur with SGA or LGA offspring, we examined trajectories after excluding women whose pregnancies had been complicated by hypertensive disorders of pregnancy (HDP), diabetes (pre-pregnancy or gestational diabetes mellitus (GDM)) or stillbirth. We performed additional analyses among women with available information on offspring birthweight in their first two pregnancies to see if recurrence of SGA or LGA impacted cardiovascular life course trajectories. In these analyses we contrasted women who had one or two of their first two pregnancies complicated by SGA or LGA against women with AGA offspring in first and second pregnancy.

Participants provided informed consent. HUNT was approved by the Regional Committee for Medical and Health Research Ethics and the Norwegian Data Inspectorate Board. This study was approved by the Central Norway Regional Committee for Medical and Health Research Ethics.

## Supplementary information

Supplementary Information.

## Data Availability

Due to restrictions imposed by the HUNT Research Centre (in accordance with Norwegian Data Inspectorate), data cannot be made publicly available. Data are currently stored in the HUNT databank, and there are restrictions in place for the handling of HUNT data files. Data used from the HUNT Study in research projects will be made available on request to the HUNT Data Access Committee (hunt@medisin.ntnu.no). The HUNT data access information (available here: http://www.ntnu.edu/hunt/data) describes in detail the policy regarding data availability.
