# Supplementary material for: Life Course Trajectories of Maternal Cardiovascular Risk Factors according to Offspring Birthweight: The HUNT Study
Source: Sci Rep. 2020 Jun 26;10:10436. doi: 10.1038/s41598-020-66365-3 (PMC7319986; doi:10.1038/s41598-020-66365-3)

Supplementary material

Life Course Trajectories of Maternal Cardiovascular Risk Factors according to Offspring Birthweight: The HUNT Study

Julie Horn; Eirin B. Haug; Amanda R. Markovitz; Abigail Fraser; Lars J. Vatten; Pål R. Romundstad; Janet W. Rich-Edwards; Bjørn O. Åsvold

Figure S1. Flow chart of study population.

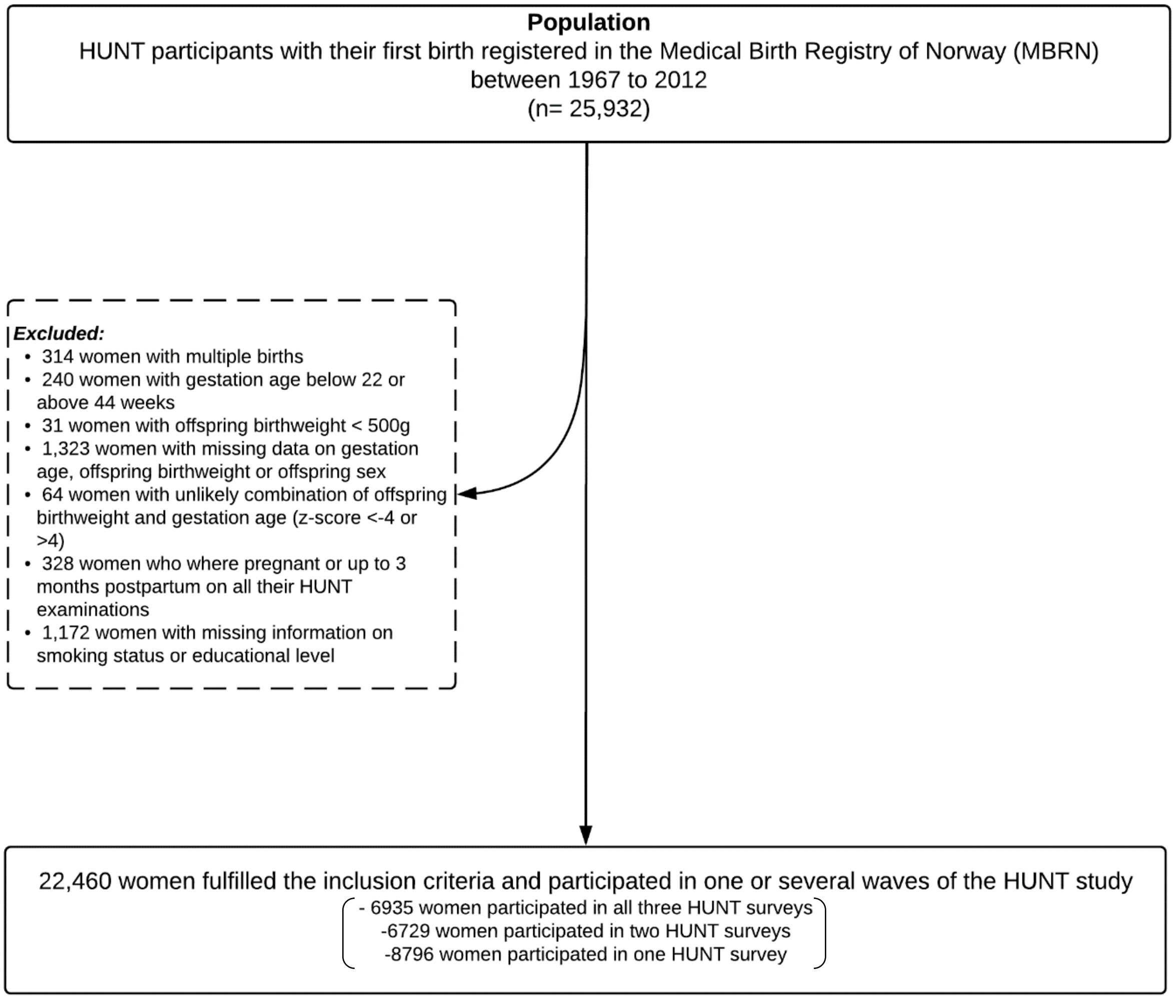

**Table S1.** Risk factor assessment by HUNT survey				
**CVD risk factor**		**HUNT1 (1984-86)**	**HUNT2 (1995-97)**	**HUNT3 (2006-08)**
Blood pressure		Measured manually two times at 1-minute intervals using a sphygmomanometer after the person had come to rest<br>The current study used the mean value of these 2 measurements.	Measured three times at 1-minute intervals using an automatic oscillometric method (Dinamap, Critikon, Florida) after the person had come to rest, cuff size adjusted to arm circumference<br>The current study used the mean value of the 2nd and 3rd measurements.	Measured three times at 1-minute intervals using an automatic oscillometric method (Dinamap, Critikon, Florida) after the person had come to rest, cuff size adjusted to arm circumference<br>The current study used the mean value of the 2nd and 3rd measurements. The 2nd measurement was used for women who lacked the 3rd measurement due to sick leave among staff.
Resting heart rate		Measured manually one time	See blood pressure measurement in HUNT2	See blood pressure measurement in HUNT3
Height and weight		Measured with the person wearing light clothes and no shoes and were rounded to the nearest cm (height) and half kilo (weight)	Measured with the person wearing light clothes and no shoes and were rounded to the nearest cm (height) and half kilo (weight)	Measured with the person wearing light clothes and no shoes and were rounded to the nearest cm (height) and half kilo (weight)
Height and weight at age 18		-	-	Self-reported
Waist circumference		-	Measured to the nearest cm while the person was standing with arms hanging down at the height of the umbilicus	Measured to the nearest cm while the person was standing with arms hanging down at the height of the umbilicus
Hip circumference		-	Measured to the nearest cm while the person was standing with arms hanging down at the thickest part of the hip	Measured to the nearest cm while the person was standing with arms hanging down at the thickest part of the hip
Triglycerides		-	Analyzed in fresh non-fasting serum samples using enzymatic colorimetric methods (Boeheringer Mannheim, Germany)	Analyzed in fresh non-fasting serum samples by a glycerol phosphate oxidase methodology, equipment from Abbott, Clinical Chemistry, USA
HDL cholesterol		-	Analyzed in fresh non-fasting serum samples using enzymatic	Analyzed in fresh non-fasting serum samples with an accelerator

			colorimetric methods (Boeheringer Mannheim, Germany)	selective detergent methodology, equipment from Abbott, Clinical Chemistry, USA
Total cholesterol		-	Analyzed in fresh non-fasting serum samples using enzymatic colorimetric methods (Boeheringer Mannheim, Germany)	Analyzed in fresh non-fasting serum samples by a cholesterol esterase methodology, equipment from Abbott, Clinical Chemistry, USA
Non-HDL cholesterol		-	Calculated as the difference between total and HDL cholesterol	Calculated as the difference between total and HDL cholesterol
Glucose		- Non-fasting capillary glucose was measured in participants above 40 years, using Reflocheck-Glucose, Boehringer Mannheim, Germany<br>- Fasting capillary glucose was measured in persons with non-fasting capillary glucose ≥8.0 mmol/L<br>- 2-hour oral glucose tolerance test was given if fasting capillary glucose was <7.0 mmol/L<br>Capillary levels were transformed to equate serum values (in mmol/L) by multiplying with 1.11	Serum glucose measured in fresh non-fasting serum samples in all participants using an enzymatic hexokinase method	Serum glucose measured in fresh non-fasting serum samples in all participants using an enzymatic hexokinase method
Diabetes mellitus		Self-report, fasting serum glucose ≥7.0 mmol/L or 2-hour post-load serum glucose ≥11.1 mmol/L	Self-report or non-fasting serum glucose ≥11.1 mmol/L	Self-report or non-fasting serum glucose ≥11.1 mmol/L
C-reactive protein (CRP)		-	Measured after 2 years of serum storage at -80 °C in a sub-sample from 4 out of 24 municipalities using a C-reactive protein ultra-sensitive assay (Tina-quant(R), Roche, Basel, Switzerland)	Measured in fresh serum samples in all participants using a latex immunoassay (Abbott, Clinical Chemistry, USA)

Table S2. Predicted mean levels of cardiovascular disease risk factors by age at follow-up in women according to weight for gestational age in first pregnancy.

	Extremely small for gestational age[a] (n=782)		Appropriate for gestational age[b] (n=21,299)	Extremely large for gestational age[c] (n=379)	
	Difference estimate (95% CI)	p-value	Predicted mean (95% CI)	Difference estimate (95% CI)	p-value
BMI (kg/m^2)					
20 years	-0.2 (-0.5, -0.1)	0.171	22.8 (22.7, 22.9)	1.2 (0.9, 1.6)	<0.001
30 years	-0.6 (-0.9, -0.2)	0.02	24.0 (24.0, 24.1)	1.6 (1.0, 2.1)	<0.001
40 years	-0.4 (-0.8, -0.1)	0.001	25.0 (24.9, 25.1)	1.5 (1.0, 2.1)	<0.001
50 years	-0.7 (-1.1, -0.3)	0.002	26.1 (26.0, 26.2)	1.6 (1.0, 2.3)	<0.001
60 years	-0.6 (-1.2, -0.04)	0.04	26.8 (26.6, 27.0)	1.6 (0.7, 2.5)	0.001
Waist circumference (cm)					
20 years	2.0 (-1.3, 5.2)	0.23	77.1 (76.4, 77.8)	2.7 (-0.7, 6.1)	0.12
30 years	-1.7 (-3.4, -0.1)	0.04	81.9 (81.5, 82.2)	4.9 (2.7, 7.1)	<0.001
40 years	-0.9 (-2.1, 0.4)	0.18	83.6 (83.4, 83.8)	3.6 (1.8, 5.3)	<0.001
50 years	-1.5 (-2.8, -0.3)	0.02	85.7 (85.4, 86.0)	2.9 (0.9, 4.9)	0.004
60 years	-0.5 (-2.1, 1.2)	0.60	87.0 (86.6, 87.5)	4.0 (1.3, 6.7)	0.004
Hip circumference (cm)					
20 years	1.1 (-1.9, 4.0)	0.48	97.9 (97.3, 98.6)	-0.1 (-3.2, 2.9)	0.93
30 years	-2.2 (-3.6, -0.8)	0.003	101.0 (100.7, 101.3)	3.8 (2.0, 5.7)	<0.001
40 years	-1.0 (-2.0, 0.03)	0.06	102.0 (101.8, 102.2)	2.7 (1.3, 4.1)	<0.001
50 years	-1.4 (-2.4, -0.5)	0.004	103.1 (102.9, 103.3)	2.3 (0.8, 3.9)	0.003
60 years	-0.6 (-1.9, 0.6)	0.33	103.1 (102.8, 103.4)	1.6 (-0.5, 3.6)	0.13
Waist to hip ratio					
20 years	0.0 (-0.01, 0.03)	0.34	0.8 (0.8, 0.8)	0.03 (0.01-0.05)	0.002
30 years	0.0 (-0.01, 0.01)	0.67	0.8 (0.8, 0.8)	0.02 (0.00-0.03)	0.01
40 years	0.0 (-0.01, 0.01)	0.69	0.8 (0.8, 0.8)	0.01 (0.00-0.02)	0.03
50 years	0.0 (-0.01, 0.00)	0.37	0.8 (0.8, 0.8)	0.01 (0.00-0.02)	0.21
60 years	0.0 (-0.01, 0.01)	0.47	0.9 (0.8, 0.9)	0.02 (0.01-0.04)	0.01
Systolic blood pressure (mmHg)					
20 years	1.4 (-1.6, 4.5)	0.35	121.0 (117.9, 124.0)	2.8 (-0.7, 6.4)	0.12
30 years	1.8 (0.2, 3.3)	0.03	119.1 (117.5, 120.7)	0.2 (-2.0, 2.4)	0.88
40 years	1.6 (0.1, 3.1)	0.04	123.2 (121.7, 124.7)	-0.4 (-2.6, 1.9)	0.74
50 years	2.4 (0.5, 4.2)	0.01	132.2 (130.4, 134.0)	1.1 (-1.9, 4.1)	0.48

60 years	4.0 (1.3, 6.8)	0.004	142.2 (139.5, 145.0)	-2.2 (-6.6, 2.4)	0.35
Diastolic blood pressure (mmHg)					
20 years	2.5 (0.3, 4.8)	0.03	70.8 (68.5, 73.1)	2.0 (-0.8, 4.7)	0.16
30 years	0.2 (-0.9, 1.3)	0.73	70.6 (69.4, 71.7)	0.5 (-1.1, 2.1)	0.54
40 years	1.8 (0.8, 2.8)	0.001	76.4 (75.4, 77.4)	-1.1 (-2.6, 0.5)	0.17
50 years	1.6 (0.4, 2.7)	0.01	80.4 (79.3, 81.6)	1.3 (-0.7, 3.2)	0.20
60 years	0.5 (-1.3, 2.2)	0.60	80.6 (78.8, 82.3)	0.2 (-2.7, 3.0)	0.92
Non-HDL cholesterol (mmol/l)					
20 years	0.3 (-0.03, 0.6)	0.07	3.2 (3.1, 3.3)	0.2 (-0.1, 0.6)	0.16
30 years	0.1 (-0.1, 0.3)	0.17	3.4 (3.4, 3.4)	0.2 (-0.02, 0.4)	0.07
40 years	0.1 (-0.1, 0.2)	0.44	3.7 (3.7, 3.8)	0.2 (-0.01, 0.3)	0.06
50 years	0.02 (-0.1, 0.1)	0.80	4.3 (4.3, 4.3)	0.2 (0.04, 0.4)	0.02
60 years	-0.1 (-0.3, 0.1)	0.29	4.8 (4.7, 4.8)	-0.03 (-0.3, 0.2)	0.82
HDL cholesterol (mmol/l)					
20 years	-0.04 (-0.2, 0.1)	0.49	1.4 (1.4, 1.5)	-0.1 (-0.2,0.02)	0.12
30 years	-0.02 (-0.1, 0.03)	0.43	1.4 (1.4, 1.4)	-0.1 (-0.1, 0.02)	0.13
40 years	0.02 (-0.02, 0.1)	0.36	1.4 (1.4, 1.4)	-0.1 (-0.1, 0.02)	0.01
50 years	0.02 (-0.02, 0.06)	0.42	1.5 (1.5, 1.5)	-0.1 (-0.1, 0.02)	0.14
60 years	0.04 (-0.01, 0.09)	0.13	1.5 (1.5, 1.6)	-0.1 (-0.1, 0.03)	0.21
Triglycerides (mmol/L)					
20 years	0.1 (-0.1, 0.3)	0.39	1.2 (1.2, 1.3)	0.02 (-0.2, 0.3)	0.85
30 years	-0.1 (-0.2, 0.1)	0.43	1.2 (1.1, 1.2)	0.1 (-0.04, 0.3)	0.13
40 years	-0.02 (-0.1, 0.1)	0.77	1.2 (1.2, 1.3)	0.2 (0.02, 0.3)	0.02
50 years	0.02 (-0.1, 0.1)	0.65	1.5 (1.4, 1.5)	0.1 (-0.04, 0.3)	0.12
60 years	-0.03 (-0.2, 0.1)	0.72	1.7 (1.6, 1.7)	0.01 (-0.3, 0.3)	0.96
Non-fasting glucose (mmol/l)					
20 years	-0.04 (-0.4, 0.3)	0.82	4.9 (4.8, 5.0)	-0.2 (-0.6, 0.2)	0.27
30 years	-0.1 (-0.3, 0.1)	0.35	4.9 (4.9, 4.9)	0.4 (0.2, 0.6)	0.001
40 years	0.02 (-0.1, 0.2)	0.77	5.1 (5.1, 5.2)	0.02 (-0.2, 0.2)	0.82
50 years	0.01 (-0.1, 0.2)	0.94	5.3 (5.3, 5.4)	0.3 (0.1, 0.6)	0.01
60 years	-0.1 (-0.4, 0.1)	0.29	5.6 (5.5, 5.6)	0.6 (0.2, 1.0)	0.002
Resting heart rate (beats/min)					
20 years	-1.5 (-4.4, 1.5)	0.33	75.5 (74.8, 76.2)	3.0 (-0.4, 6.5)	0.09
30 years	1.6 (0.2, 3.1)	0.03	74.0 (73.7, 74.3)	-0.5 (-2.5, 1.5)	0.60
40 years	0.2 (-1.0, 1.4)	0.79	73.7 (73.4, 73.9)	0.9 (-0.9, 2.7)	0.34
50 years	-0.5 (-1.9, 0.8)	0.44	73.1 (72.8, 73.4)	-1.4 (-3.6, 0.9)	0.22

60 years	0.6 (-1.4, 2.6)	0.56	73.0 (72.4, 73.5)	0.8 (-2.5, 4.1)	0.64
CRP[d] (mg/l)					
20 years	1.0 (0.5, 2.5)	0.75	1.7 (1.4, 1.9)	2.0 (0.8, 4.7)	0.12
30 years	1.2 (0.8, 2.0)	0.39	1.0 (0.9, 1.1)	1.4 (0.9, 2.2)	0.10
40 years	0.9 (0.7, 1.2)	0.64	0.8 (0.8, 0.9)	1.2 (0.9, 1.6)	0.20
50 years	0.9 (0.8, 1.1)	0.37	1.0 (0.9, 1.0)	1.1 (0.8, 1.5)	0.57
60 years	1.1 (0.9, 1.3)	0.37	1.3 (1.2, 1.4)	1.2 (0.9, 1.6)	0.19

CI=confidence interval; BMI=body mass index; HDL=high density lipoprotein; CPR=C-reactive protein

[a] Extremely small for gestational age (SGA) was defined as birthweight below the 3rd centile for the Norwegian population

[b] Appropriate for gestational age (AGA) was defined as birthweight ≥ 3rd and ≤ 97th centile for the Norwegian population

[c] Extremely large for gestational age (LGA) was defined as birthweight above the 97th centile for the Norwegian population

[d] CRP is given as geometric mean values where the difference equates to the ratio of geometric mean CRP between women with SGA or LGA offspring and women with AGA offspring.

Table S3. Predicted mean levels of cardiovascular disease risk factors by age at follow-up in 20,757 women with uncomplicated[a] first pregnancies according to birthweight for gestational age.

	**Small for gestational age[b] (n=2,674)**		**Appropriate for gestational age[c] (n=16,822)**	**Large for gestational age[d] (n=1,261)**	
	Difference estimate	p-value	Predicted mean	Difference estimate	p-value
BMI (kg/m^2)					
20 years	-0.4 (-0.5, -0.2)	<0.001	22.7 (22.6, 22.8)	1.0 (0.9, 1.2)	<0.001
30 years	-0.6 (-0.8, -0.4)	<0.001	23.9 (23.8, 24.0)	1.2 (1.0, 1.5)	<0.001
40 years	-0.8 (-1.0, -0.6)	<0.001	24.9 (24.8, 25.0)	1.3 (1.0, 1.6)	<0.001
50 years	-0.8 (-1.1, -0.6)	<0.001	25.9 (25.8, 26.1)	1.3 (1.0, 1.7)	<0.001
60 years	-0.9 (-1.2, -0.5)	<0.001	26.7 (26.5, 26.8)	1.4 (0.9, 1.9)	<0.001
Waist circumference (cm)					
20 years	-1.1 (-2.7, 0.5)	0.18	76.8 (76.1, 77.6)	2.6 (0.8, 4.3)	0.004
30 years	-0.7 (-1.6, 0.2)	0.15	81.3 (80.9, 81.7)	3.5 (2.3, 4.7)	<0.001
40 years	-1.8 (-2.5, -1.1)	<0.001	83.3 (83.0, 83.6)	2.9 (2.0, 3.9)	<0.001
50 years	-1.9 (-2.6, -1.2)	<0.001	85.5 (85.2, 85.8)	2.1 (1.1, 3.2)	<0.001
60 years	-1.8 (-2.7, -0.8)	<0.001	86.9 (86.5, 87.4)	3.5 (2.0, 5.1)	<0.001
Hip circumference (cm)					
20 years	-1.8 (-3.3, -0.3)	0.02	97.9 (97.2, 98.5)	2.1 (0.5, 3.7)	0.01
30 years	-1.0 (-1.8, -0.2)	0.01	100.5 (100.2, 100.8)	2.8 (1.7, 3.8)	<0.001
40 years	-1.8 (-2.3, -1.2)	<0.001	101.9 (101.7, 102.1)	2.3 (1.5, 3.1)	<0.001
50 years	-1.3 (-1.9, -0.8)	<0.001	103.0 (102.7, 103.2)	1.5 (0.7, 2.3)	<0.001
60 years	-1.5 (-2.2, -0.8)	0.001	103.1 (102.7, 103.4)	1.2 (0.1, 2.4)	0.03
Waist to hip ratio					
20 years	0.0 (-0.01, 0.01)	0.41	0.8 (0.8, 0.8)	0.01 (0.00-0.02)	0.03
30 years	0.0 (-0.01, 0.01)	0.90	0.8 (0.8, 0.8)	0.01 (0.01-0.02)	0.001
40 years	0.0 (-0.01, 0.00)	0.04	0.8 (0.8, 0.8)	0.01 (0.00-0.02)	0.001
50 years	-0.01 (-0.01, 0.00)	0.001	0.8 (0.8, 0.8)	0.01 (0.00-0.01)	0.02
60 years	0.0 (-0.01, 0.01)	0.50	0.8 (0.8, 0.8)	0.02 (0.01-0.03)	<0.001
Systolic blood pressure (mmHg)					
20 years	0.7 (-1.0, 2.3)	0.42	119.0 (118.2, 119.8)	0.7 (-1.3, 2.6)	0.49
30 years	0.8 (-0.1, 1.7)	0.08	116.8 (116.4, 117.2)	0.1 (-1.1, 1.3)	0.89
40 years	1.0 (0.2, 1.8)	0.02	121.0 (120.6, 121.3)	-0.2 (-1.4, 1.1)	0.80

50 years	1.6 (0.6, 2.7)	0.003	129.0 (128.5, 129.4)	1.3 (-0.3, 3.0)	0.11
60 years	1.5 (-0.2, 3.1)	0.08	137.5 (136.7, 138.3)	-0.4 (-3.0, 2.1)	0.75
Diastolic blood pressure (mmHg)					
20 years	0.7 (-0.6, 1.9)	0.30	68.0 (67.4, 68.6)	-0.01 (-1.5, 1.5)	0.99
30 years	-0.3 (-0.9, 0.4)	0.41	70.0 (69.7, 70.2)	0.7 (-0.2, 1.6)	0.13
40 years	0.7 (0.1, 1.3)	0.01	74.2 (74.0, 74.4)	-0.02 (-0.9, 0.8)	0.96
50 years	0.7 (0.02, 1.4)	0.06	78.5 (78.2, 78.8)	0.9 (-0.1, 2.0)	0.09
60 years	-0.2 (-1.2, 0.8)	0.69	80.0 (79.5, 80.5)	-0.2 (-1.8, 1.4)	0.82
Non-HDL cholesterol (mmol/l)					
20 years	0.1 (-0.1, 0.2)	0.44	3.2 (3.1, 3.2)	0.04 (-0.1, 0.2)	0.66
30 years	0.03 (-0.1, 0.1)	0.53	3.4 (3.3, 3.4)	0.1 (0.01, 0.3)	0.05
40 years	-0.01 (-0.1, 0.1)	0.68	3.7 (3.7, 3.7)	0.1 (0.01, 0.2)	0.02
50 years	-0.04 (-0.1, 0.03)	0.26	4.3 (4.3, 4.3)	0.1 (0.03, 0.3)	0.01
60 years	-0.03 (-0.1, 0.1)	0.61	4.8 (4.7, 4.8)	0.2 (-0.01, 0.3)	0.05
HDL cholesterol (mmol/l)					
20 years	-0.02 (-0.1, 0.04)	0.58	1.4 (1.4, 1.5)	-0.1 (-0.1, 0.0)	0.07
30 years	0.01 (-0.02, 0.1)	0.35	1.4 (1.4, 1.4)	-0.02 (-0.1, 0.02)	0.30
40 years	0.02 (-0.01, 0.04)	0.08	1.4 (1.4, 1.4)	-0.03 (-0.1, 0.00)	0.09
50 years	0.01 (-0.02, 0.03)	0.69	1.5 (1.5, 1.5)	-0.02 (-0.1, 0.02)	0.36
60 years	0.03 (-0.01, 0.1)	0.08	1.6 (1.5, 1.6)	-0.05 (-0.1, 0.00)	0.04
Triglycerides (mmol/l)					
20 years	0.04 (-0.1, 0.2)	0.52	1.2 (1.2, 1.3)	0.03 (-0.1, 0.2)	0.64
30 years	0.01 (-0.1, 0.1)	0.73	1.1 (1.1, 1.2)	0.1 (-0.02, 0.2)	0.14
40 years	0.01 (-0.04, 0.1)	0.63	1.2 (1.2, 1.3)	0.1 (0.01, 0.2)	0.03
50 years	0.03 (-0.03, 0.1)	0.38	1.4 (1.4, 1.5)	0.1 (-0.03, 0.2)	0.15
60 years	-0.02 (-0.1, 0.1)	0.67	1.7 (1.6, 1.7)	0.1 (-0.02, 0.3)	0.10
Non-fasting glucose (mmol/l)					
20 years	-0.00 (-0.2, 0.2)	0.98	4.8 (4.8, 4.9)	0.03 (-0.1, 0.2)	0.69
30 years	-0.06 (-0.2, 0.04)	0.23	4.9 (4.8, 4.9)	0.2 (0.1, 0.3)	0.01
40 years	0.00 (-0.1, 0.1)	0.95	5.1 (5.1, 5.1)	0.04 (-0.1, 0.1)	0.50
50 years	-0.1 (-0.1, 0.02)	0.16	5.3 (5.3, 5.4)	0.1 (0.04, 0.2)	0.19
60 years	-0.1 (-0.2, 0.1)	0.21	5.6 (5.5, 5.6)	0.2 (-0.03, 0.4)	0.09
Resting heart rate (beats/min)					
20 years	-0.5 (-2.0, 1.1)	0.57	75.0 (74.3, 75.8)	-0.2 (-2.1, 1.7)	0.83
30 years	0.6 (-0.2, 1.4)	0.14	73.9 (73.5, 74.2)	-0.9 (-2.0, 0.2)	0.11
40 years	-0.2 (-0.8, 0.5)	0.67	73.7 (73.4, 74.0)	-0.9 (-1.9, 0.1)	0.08

50 years	0.2 (-0.6, 1.1)	0.55	72.9 (72.6, 73.3)	-0.4 (-1.6, 0.9)	0.54
60 years	0.4 (-0.8, 1.6)	0.54	73.1 (72.5, 73.6)	0.1 (-1.8, 2.0)	0.91
CRP[e] (mg/l)					
20 years	0.9 (0.7, 1.3)	0.73	1.7 (1.4, 2.0)	0.8 (0.5, 1.3)	0.40
30 years	1.0 (0.8, 1.3)	0.88	1.0 (0.9, 1.1)	1.5 (1.1, 1.9)	0.003
40 years	0.9 (0.8, 1.1)	0.23	0.8 (0.8, 0.9)	1.0 (0.8, 1.2)	0.83
50 years	1.0 (0.9. 1.2)	0.63	1.0 (0.9, 1.0)	1.1 (0.9, 1.2)	0.69
60 years	1.1 (0.9, 1.2)	0.41	1.3 (1.2, 1.3)	1.2 (1.0, 1.5)	0.02

CI=confidence interval; BMI=body mass index; HDL=high density lipoprotein; CPR=C-reactive protein

[a] Defined as pregnancies not complicated by hypertensive disorders of pregnancy, pre-pregnancy, gestational diabetes mellitus, or stillbirth.

[b] Small for gestational age (SGA) was defined as birthweight below the 10th centile for the Norwegian population

[c] Appropriate for gestational age (AGA) was defined as birthweight ≥ 10th and ≤ 90th centile for the Norwegian population

[d] Large for gestational age (LGA) was defined as birthweight above the 90th centile for the Norwegian population

[e] CRP is given as geometric mean values where the difference equates to the ratio of geometric mean CRP between women with SGA or LGA offspring and women with AGA offspring.

Figure S2. Predictied probabilities of hypertension, obesity and diabetes according to offspring birthweight for gestational age.

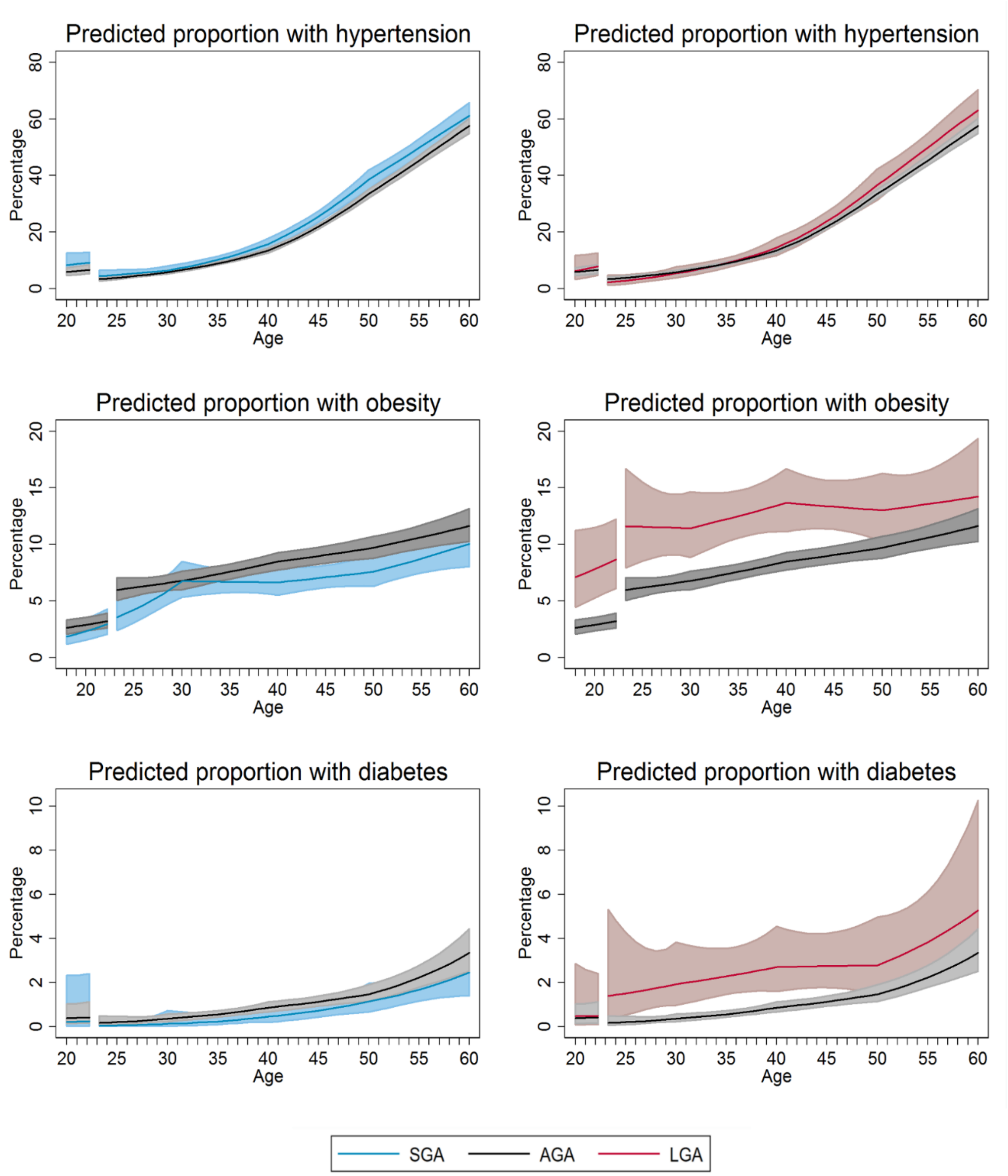

Figure S3. Life course trajectories of mean systolic blood pressure, diastolic blood pressure, resting heart rate, and CRP for women according to weight for gestational age in first and second pregnancy.

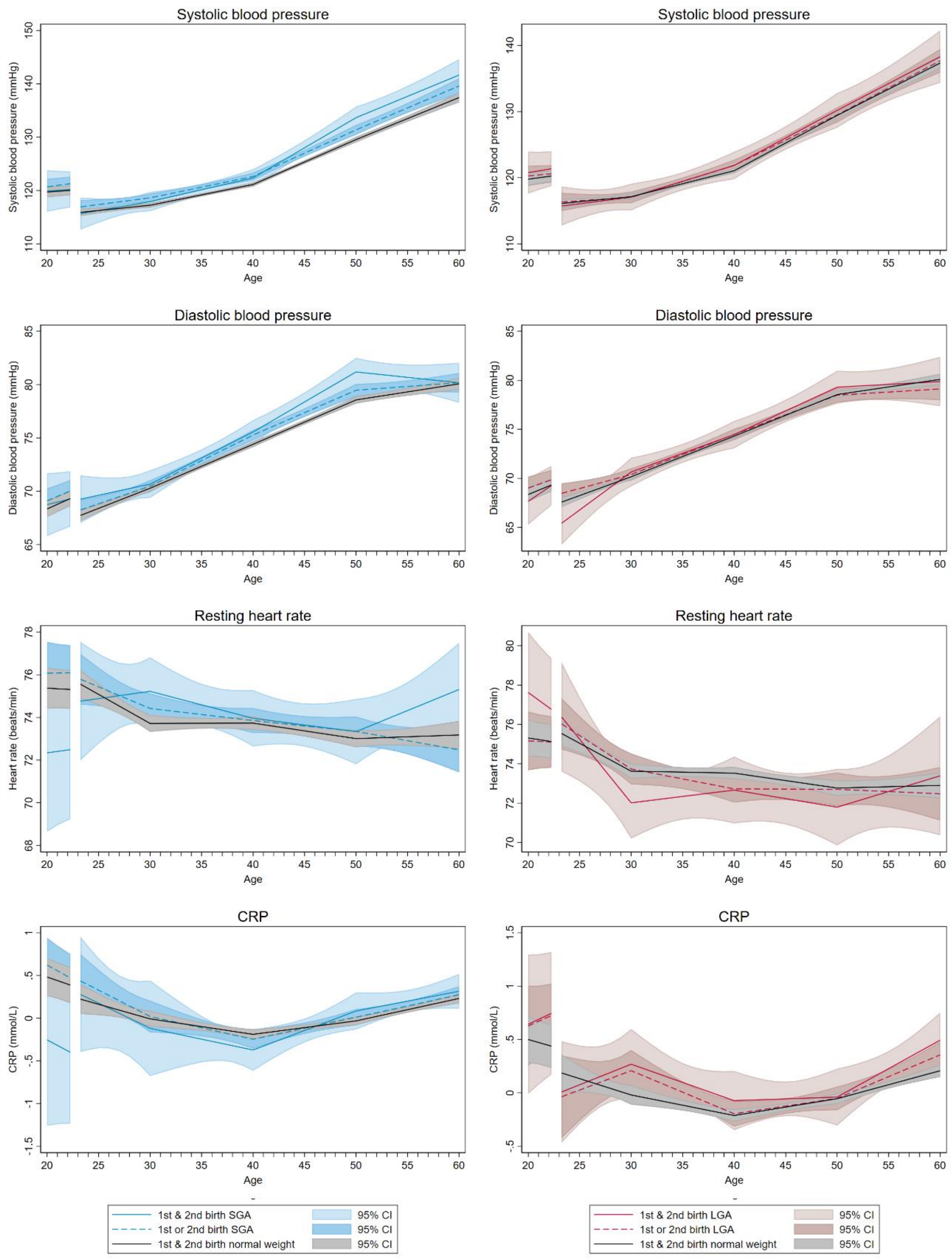

Figure S4. Life course trajectories of mean BMI, waist circumference, hip circumference, and waist to hip ratio according to weight for gestational age in first and second pregnancy.

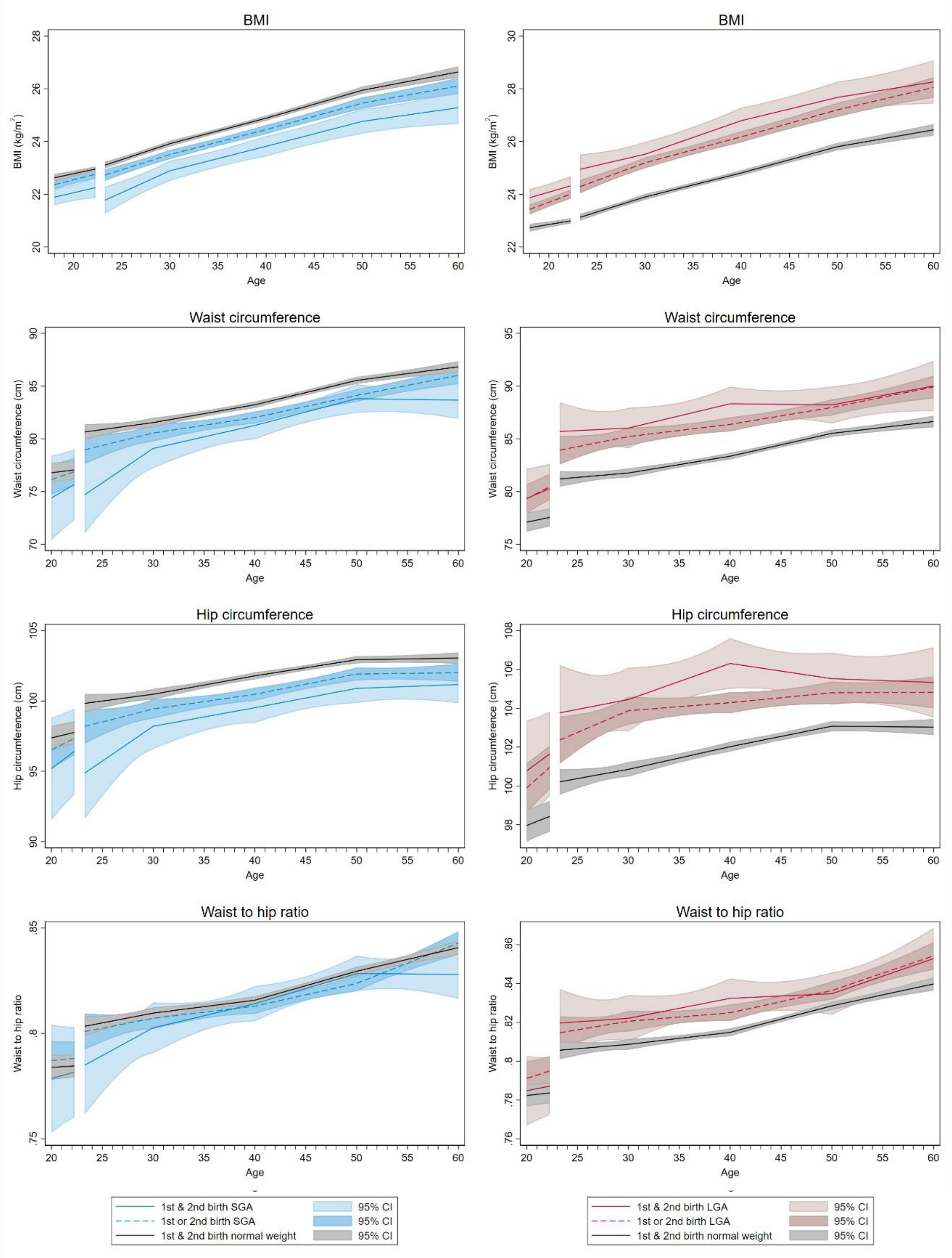

Figure S5. Life course trajectories of mean nonfasting serum Non-HDL and HDL cholesterol, triglycerides, and glucose for women according to weight for gestational age in first and second pregnancy.

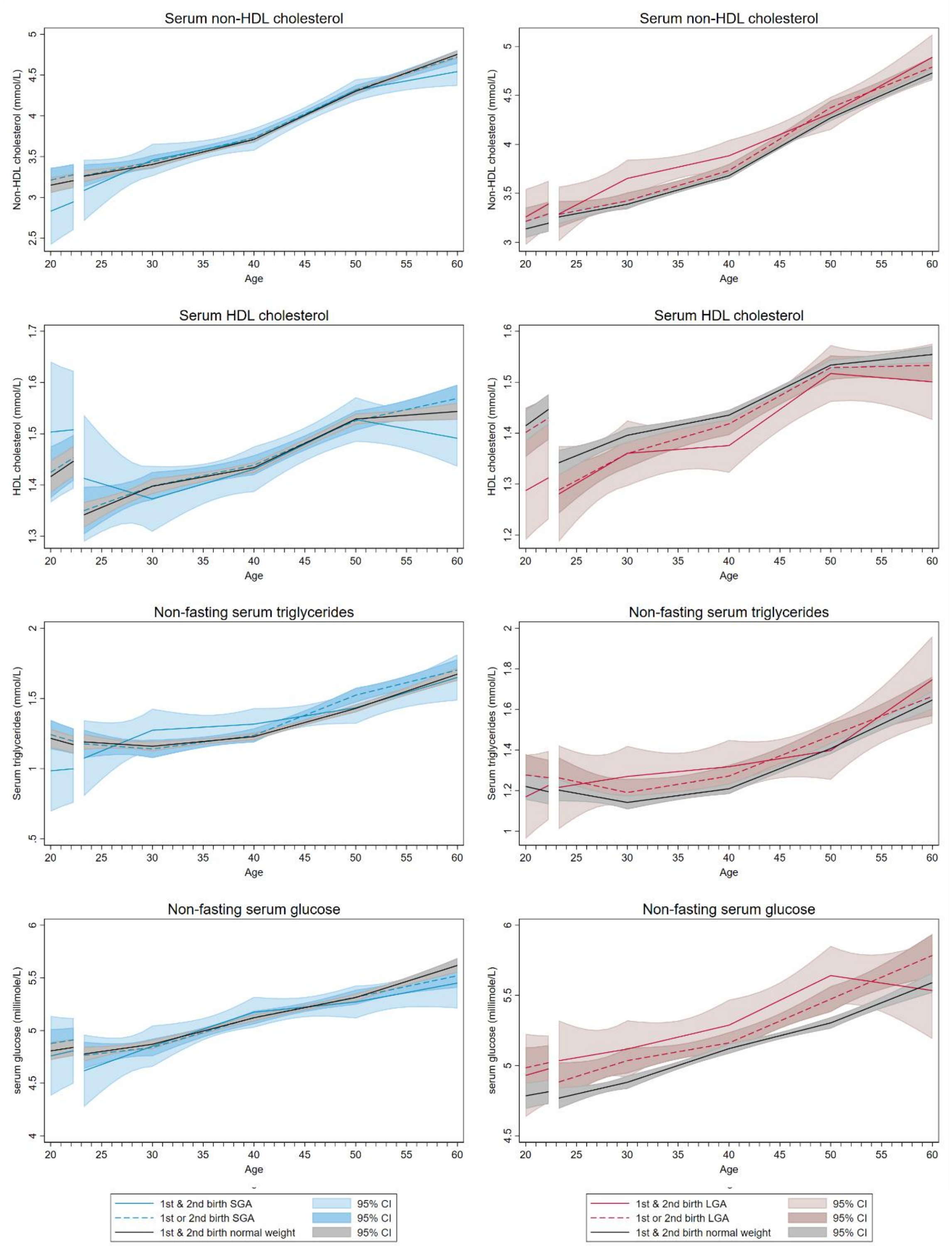

Figure S6. Number of cardiovascular risk factor measurements according to time since first birth (years) and HUNT survey.

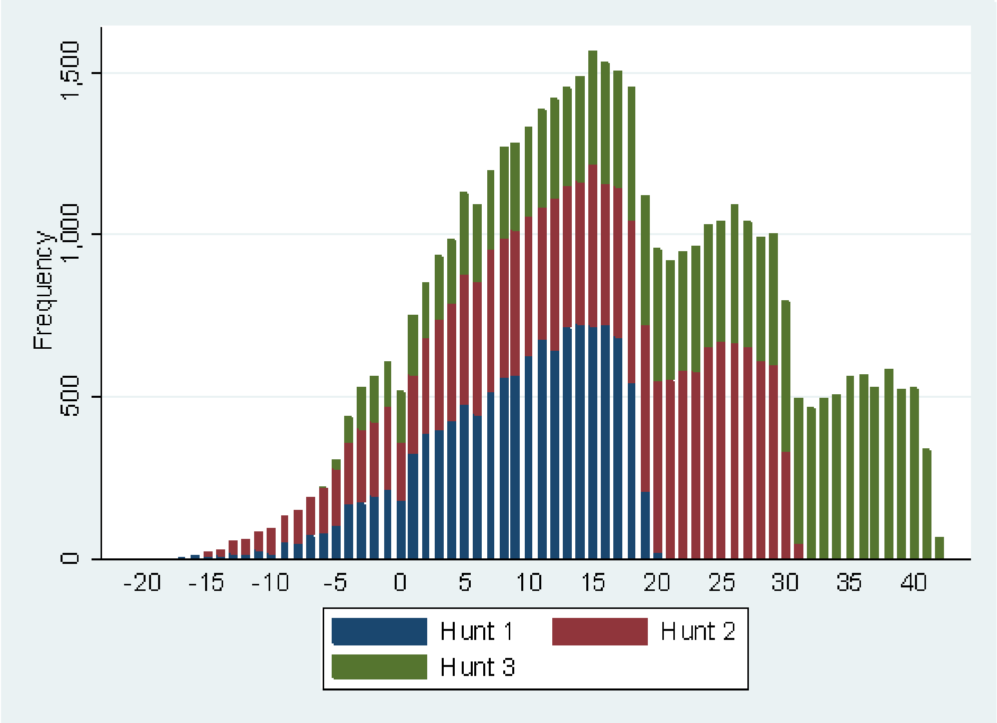

Supplement: Supplementary file 1 — Supplementary Information. [file 41598_2020_66365_MOESM1_ESM.pdf]
